# Knowledge of Hepatitis B Vaccine among Operating Room Personnel in Nigeria and Their Vaccination Status

**DOI:** 10.1155/2011/157089

**Published:** 2011-10-19

**Authors:** Emeka B. Kesieme, Kenechi Uwakwe, Eshiobo Irekpita, Andrew Dongo, Kefas John Bwala, Bamidele J. Alegbeleye

**Affiliations:** ^1^Department of Surgery, Irrua Specialist Teaching Hospital, PMB 8, Irrua, Nigeria; ^2^Department of Community Medicine, Imo State University Teaching Hospital, PMB 8, Orlu, Nigeria; ^3^Department of Surgery, University of Maiduguri Teaching Hospital, PMB 1414, Maiduguri, Nigeria; ^4^Department of Surgery, University College Hospital, PMB 5017, Ibadan, Nigeria

## Abstract

*Background*. Hepatitis B virus (HBV) infection is a well recognised occupational health hazard preventable by vaccination. *Objectives*. To determine the knowledge of operating room personnel (ORP) in Nigeria about the Hepatitis B vaccine, their perception of Hepatitis B vaccination and vaccination status against HBV. *Methods*. Four university hospitals were selected by simple random sampling. A structured questionnaire was administered to 228 ORP after obtaining consent. *Result*. Only 26.8% of ORP were vaccinated against HBV. The primary reason for not being vaccinated or for defaulting from vaccination was lack of time. Differences in age, sex, duration of practice and respondent's institution between vaccinated and unvaccinated ORP were not significant (*P* > 0.05). The majority (86.8%) had the awareness of the existence of Hepatitis B vaccine. 83.8% of respondents believed that the vaccine should be given to the ORP as part of work place safety measures. The majority were aware of the modes of transmission of HBV infection. 78.9% of respondents believed that Hepatitis B vaccine is safe and 81.1% would recommend it to another staff. *Conclusion*. Despite a good knowledge about HBV infection and vaccine, most of ORP are still not vaccinated. Hepatitis B vaccination should be a prerequisite for working in the theatre, hence putting surgical patients at reduced risk.

## 1. Introduction

The most serious occupational health hazard faced by health care workers worldwide is exposure to blood-borne pathogens. These blood-borne pathogens are mainly Hepatitis B, C, and HIV infection.

Hepatitis B is by far the most dreaded with over 2 billion people affected worldwide and 350 million people suffering from chronic hepatitis B virus infection [1]. It is more infectious than the other blood-borne pathogens and estimates of the risk of a single needlestick injury indicate a risk of 300 hepatitis B virus infection (30% risk), 30 hepatitis C virus infection (3% risk) and 3 HIV infection (0.3% risk), per 1,000 respective exposures [2].

In the United States, the incidence of HBV infection among all health care workers is estimated to be 3.5 to 4.6 infections per 1000 workers, which is 2- to 4-times the level for the general population [3]. The disease is thus more likely in health workers in Nigeria, a country with high prevalence of the disease.

Among health care workers, operating room personnel are at a high risk of infection with blood-borne pathogens through blood contact [4]. This group of health workers has been shown not to follow standard precautions and not to report all percutaneous injuries [4]. Unfortunately, researchers have also not shown enough interest in evaluating their knowledge of hepatitis B virus infection or the vaccine. Most previous studies in health care workers in developing countries have revealed inadequate knowledge of hepatitis B virus infection and inadequate practice of preventive measures against the disease [5–7].

Percutaneous injury is the most efficient mode of hepatitis B transmission among operating room personnel. The risk of developing serologic evidence of HBV infection was 37% to 62% if the blood was positive for both hepatitis B surface antigen (HBsAg) and hepatitis B e antigen (HBeAg) and 23% to 37% if the blood was positive for HBsAg and negative for HBeAg [8].

The most effective and feasible means of preventing HBV infection is by vaccination and avoidance of exposure to blood. The vaccine was initially by means of a plasma-derived HBsAg subunit which has largely been replaced by recombinant vaccine. This vaccine has been available since 1982 and has generally been described as safe and effective with a protective efficiency of 90–95% [9].

Complete vaccination against hepatitis B is achieved by administration of a three-dose regimen, with the second and third doses being given one and six months after the initial dose. A test for hepatitis B surface antibody (HBsAb) should be carried out 6–8 weeks following the final dose of the primary course of vaccination [10]. Antibody levels of over 100 miu/mL indicate a good response to vaccination. Antibody levels between 10 and 100 miu/mL indicate a poor response and a booster dose should be given immediately to improve response. A blood test should be carried out 6–8 weeks after the booster dose to check response [10].

We aimed to determine, in operating room personnel, their knowledge of the HBV vaccine, perception of vaccination, and understanding of risk factors for HBV infection.

To the best of our knowledge, no similar study has been carried out among operating room personnel in Nigeria.

## 2. Methodology

### 2.1. Study Design

Four university hospitals were selected for the study by simple random sampling. The hospitals included University of Maiduguri Teaching Hospital, Imo State University Teaching Hospital, University College Hospital, Ibadan and Irrua Specialist Teaching Hospital representing the Northern, Eastern, Western, and Southern Nigeria, respectively. All the institutions are tertiary referral centres for training undergraduate and postgraduate students and for research. All the operating theatre staff including surgeons, anaesthetists, scrub nurses, and auxiliary staff were eligible for the study.

### 2.2. Enrolment and Data Collection

After obtaining informed consent, the participating theatre staff completed a questionnaire which was based mainly on the knowledge and practice of hepatitis B immunization, knowledge of risk of exposure, and practice of postexposure prophylaxis. These questionnaires were distributed and filled in the operating room to ensure the participation of most active operating room personnel.

The following information was requested: demographic and professional characteristics, duration of work experience, status of immunization, perception of hepatitis B vaccine, and their attitude towards recommending hepatitis B vaccine to other theatre users. Awareness of various risks for exposure to blood-borne pathogens and practice of postexposure prophylaxis was explored.

Vaccinated respondents were classified into (a) those who had completed course of vaccination and the antibody test indicating a good response or (b) those who had completed a course of vaccination but defaulted on antibody test after vaccination. Staff who had never been vaccinated or started the course of vaccination but defaulted were considered to be not vaccinated.

### 2.3. Statistical Analysis

The data from all the returned questionnaires were entered into SPSS, version 16, and analysed. Both descriptive and inferential statistics were computed. The level of significance was set at *P* < 0.05.

## 3. Results

A total of 228 operating room personnel participated in the study. The majority of staff that participated in the study were surgical residents (trainee surgeons; 32%). Consultants, house surgeons, and nurses accounted for 12.7%, 10.5%, and 21% of respondents, respectively. Others included anaesthetists (12.3%), technicians (5.7%), and others (6.6%). Males accounted for 67.5% while females accounted for 32.5%. 

Sixty-one respondents (26.8%) had been vaccinated while 167 respondents had not been vaccinated (Table 1). Of the 167 respondents that had not been vaccinated, 103 (61.7%) had not commenced vaccination, 55 (32.9%) started vaccination and defaulted and 9 (5.4 %) were in the process of receiving vaccinations and awaiting further courses. Among those that were completely vaccinated, 29 respondents had undergone antibody testing which showed good response to vaccination whereas 32 respondents defaulted on antibody test after vaccination.

Of the respondents that had been vaccinated, 39 were males (63.9%) while 22 (36.1%) were females. Respondents practising for 15–20 years had not been not vaccinated. Differences in age, sex, duration of practice, and respondent's institution between the vaccinated and unvaccinated ORP were not statistically significant (*P* > 0.05).

One hundred and forty-seven (64.5%) respondents of the ORP that had not been vaccinated responded to the reasons for not being vaccinated or for defaulting while on a vaccination schedule. The primary reasons were mainly lack of time to attend (33.9%), not enough information on the vaccination (19.7%), and no idea about existence of immunization (15%).

Regarding knowledge of hepatitis B vaccine, 86.8% of respondents were aware of the existence of the vaccine (Table 2). All surgeons (including trainees) were aware of this vaccine, but 4 anaesthetists, 9 nurses, and 12 other staff were unaware of the vaccine (*P* < 0.05).

The majority (83.3%) were of the opinion that the vaccine should be given to operating room personnel as part of work place safety measure while 44.9% thought that hepatitis B vaccine can be administered simultaneously with hepatitis B immunoglobulin (HBIG) when indicated (Table 2). Most respondents also rightly indicated that complete vaccination does not consist of just 2 doses of the vaccine. Out of these 71 surgical staff, less than half (47%) could correctly state the intervals between the doses.

The majority had good knowledge of the risk factors for HBV infection. Some 91.7%, 83.2%, and 84.6% believed that hepatitis B virus infection can be transmitted through percutaneous injury, mucous membrane contact with blood, and contact of abraded skin with potentially infected tissue, respectively. Seventy-eight point three percent (78.3%) of respondents thought that they were at a greater risk of becoming infected with HBV than the general population (Table 3).

Regarding the perception of the hepatitis B vaccine, 76.8% of the respondents agreed that the vaccine is safe. Eighty-one point one percent (81.1%) of respondents would recommend the vaccine to another surgical staff. The relationship between recommending the vaccine to a colleague and perception about the safety of hepatitis virus vaccine was found to be statistically significant (*P* < 0.05). Seventy-two point one percent (72.1%) of respondents did not know how many of their colleagues that had received hepatitis B vaccine (Table 4).

## 4. Discussion

In developed countries like the United States, not only are there a significant percentage of health care workers (75%) vaccinated against hepatitis B virus infection, but plans already exist to achieving 98% hepatitis B vaccination coverage among HCWs, providing a benchmark for the elimination of occupationally acquired HBV infection [11]. Seventy-nine percent (79%) of HCWs in Sweden had received at least one dose of vaccine, but only 40% were reported to be fully vaccinated [12] while vaccination coverage was found to be 48.2% in dental workers in Japan [13]. The story has been different in Nigeria [6, 14].

This study has clearly shown a poor vaccination status (26.8%) among operating theatre personnel, a subset of health care workers obviously at high risk of occupational exposure to HBV. This is very disturbing as previous studies in the country have consistently shown low vaccination status among health care workers. Adebamowo et al. and Sofola et al. reported that only 18.1% and 37.9% of their respondents were reported to be fully vaccinated against hepatitis B infection in 1998 and 2007, respectively [6, 14]. However studies done in India have revealed better vaccination status [15, 16]. Sukriti et al. reported that only 28% HCWs in India were unvaccinated and 17% were unaware of their vaccination status [15]. Singhal et al. reported that 56.5% of health care workers were vaccinated, and in 79% of those vaccinated persons, protective levels (>10 IU/mL) of anti-hepatitis B surface antigen (anti-HBs) were seen [16].

We found that many of the staff were unvaccinated due to lack of time to attend to vaccination and not having enough information about hepatitis B vaccine. Other workers have found busy schedule, lack of knowledge about severity and vaccine efficiency, perception of low-risk status as reasons for unvaccination, refusal of vaccine due to fear of getting hepatitis B from the vaccination, and fear of side effects [7, 17, 18]. Kamolratanakul et al. found out that lack of knowledge and negative attitudes were the main reasons for refusal, in a vaccination programme against hepatitis B virus [19]. These were found to improve significantly after the dissemination of information, with acceptance rates increasing from 56.9% to 77.7% [19]. The acceptance of hepatitis B vaccine has been found to be strongly related to social influence (physicians, supervisors, role models, friends, and spouse) and knowledge of the disease and vaccine whereas refusal has been found to be primarily related to concern about vaccine side effects and problems with vaccine access [20].

Our study revealed that most of the respondents had good knowledge of the risk factors for hepatitis B virus infection. This contrasts others that have demonstrated a very low knowledge of hepatitis B infection and poor safe practices to prevent transmission of the infection [6, 7, 21]. We think that the reason for this is that the majority of our respondents were doctors or nurses and so are more educated than the lower cadre of health care workers. This fact is, however, substantiated by a study by Ibekwe and Ibeziako in which there was a significant occupational difference in perception to an increased risk of contracting hepatitis B virus infection with only 5.5% of the ward attendants as against 67.9% of other workers feeling that their jobs exposed them to increased risk [22].

Our study revealed that a significant percentage of operating room personnel believed the vaccine to be safe and will recommend it to another colleague. This may be attributable to their good knowledge of the vaccine and risk factors for HBV infection. Perception of vaccine safety has been identified as the most important predictor for acceptance and for willingness to recommend HBV vaccination to other heath care workers. Vaccinated HCWs were more likely to recommend vaccination to other healthcare personnel as were those younger than 40 years of age [23].

One of the important limitations of this study is its reliance on information obtained from the respondents about their vaccination status. There is a possibility of the respondents overreporting vaccination; hence, we may have overestimated the proportion of “adequately” vaccinated respondents. A second limitation is that we asked about the total number of HBV vaccine injections received by the respondent, without considering the time intervals between these injections. The third limitation is that we assumed that all the respondents who had received 3 doses are adequately vaccinated with or without undergoing post-vaccination antibody assay. Five (5%) of individuals have been found not to respond to the initial vaccination series, and this subset of individuals should undergo a second series of three vaccinations [24].

## 5. Conclusion

Hepatitis B virus infection is an important disease, preventable by vaccination and compliance with universal precautions. The vaccination status of the theatre staff in Nigeria is low and unacceptable especially with the availability of the vaccine. To protect the patient, complete HBV vaccination should be a prerequisite for theatre staff to commence work. Programmes designed to increase awareness relating to HBV infection, hepatitis B vaccine, and adherence to universal precautions are required to improve vaccination status.

## Figures and Tables

**Table 1:** Vaccination status.

Vaccination status	
Vaccinated	61 (26.8%)
Not vaccinated	167 (73.2%)
Total	228 (100%)

**Table 2:** Knowledge of hepatitis B vaccine.

Are you aware of vaccination for hepatitis B	
Yes	198 (86.8%)
No	30 (13.2%)
Total	228 (100%)

Hepatitis B vaccine should be given to ORP as part of work place safety	

Yes	191 (83.8%)
No	13 (5.7%)
DNK	24 (10.5%)
Total	228 (100%)

Hepatitis B vaccine can be administered simultaneously with HBIG (the immunoglobulin) when indicated	

Yes	102 (44.9%)
No	36 (15.9%)
DNK	89 ( 39.2%)
Total	227(100%)

When indicated as part of PEP, it should be administered within 24 hrs of exposure	

Yes	116 (50.9%)
No	27 (11.8%)
DNK	85 (37.3%)
Total	227 (100%)

For complete protection, it consists of two doses at 0 and 6 months	

Yes	55 (24.6%)
No	71 (31.7%)
DNK	98 (43.8)
Total	224 (100%)

**Table 3:** Knowledge of risk factors.

Percutaneous injury with blood	
Yes	188 (91.7%)
No	17 (8.3%)
Total	205 (100%)

Mucous membrane contact with blood	

Yes	168 (83.2%)
No	33 (16.8%)
Total	197 (100%)

Contact of abraded skin with potentially infected tissue	

Yes	170 (84.6%)
No	31 (15.4%)
Total	201 (100%)

Contact of skin afflicted with dermatitis with potentially infected body fluid	

Yes	146 (78.1%)
No	41 (21.9%)
Total	187 (100%)

Do you agree you are at risk more than the general population	

Agree	173 (78.3%)
Disagree	27 (12.2%)
DNK	21 (9.5%)
Total	221 (100%)

**Table 4:** Perception of HBV vaccine.

Do you agree that HBV vaccine is safe	
Yes	180 (78.9%)
No	14 (6.1%)
DNK	34 (14.9%)
Total	228 (100%)

I will recommend HBV vaccine to another surgical staff	

Yes	185 (81.1%)
No	16 (7.0%)
DNK	27 (11.8%)
Total	224 (100%)

How many of your colleagues do you think have received HBV vaccination	

Yes	29 (15.8%)
No	22 (12.0%)
DNK	132 (72.1%)
Total	183 (100%)
